# Changes in hyperglycaemia-related testing for prediabetes and type 2 diabetes mellitus management: a prospective, cross-sectional survey of 16 years of general practice data from Australia

**DOI:** 10.1186/s12875-022-01896-4

**Published:** 2022-11-21

**Authors:** Andrew Leigh, Jennifer Hunter, Christopher Harrison, Helena Britt, Eugen Molodysky

**Affiliations:** 1 The University of Sydney, Sydney, Faculty of Medicine and Health, Sydney School of Medicine, Anderson Stuart Building, The University of Sydney, Camperdown, NSW 2006 Australia; 2 Health Research Group, PO Box 910, Bondi Junction, NSW 3155 Australia; 3 The University of Sydney, Faculty of Medicine and Health, Sydney School of Public Health, Menzies Centre for Health Policy and Economics, Charles Perkins Centre (D17), The University of Sydney, Camperdown, NSW 2006 Australia; 4 The University of Sydney, Faculty of Medicine and Health, Sydney School of Public Health, Edward Ford Building, A27 Fisher Rd, University of Sydney, Camperdown, NSW 2006 Australia

**Keywords:** General Practice, Primary Care, Diabetes Mellitus Type 2, Prediabetes, Insulin Resistance, Health Care Survey

## Abstract

**Background:**

The rising prevalence of prediabetes increases the population risk of type 2 diabetes mellitus (T2DM), metabolic syndrome and cardiovascular disease. Early identification by General Practitioners (GPs) provides opportunities for lifestyle modifications that can lower these risks.

**Methods:**

This study examined 16 years of hyperglycaemia-related testing for patients in Australia aged 13 years or older with, or at risk of a diagnosis of T2DM. The Bettering the Evaluation and Care of Health (BEACH) study is a national cross-sectional survey, with a single-stage, cluster sampling design. Approximately 1,000 GPs were randomly selected annually (2000/01–20,015/16) from across Australia, who each recorded details of 100 consecutive clinical encounters with consenting patients. Point estimates were adjusted for intracluster correlation and GP characteristics.

**Results:**

Fifteen thousand six hundred seventy nine GPs recorded details of 1,387,190 clinical encounters with patients aged 13 + years. Prediabetes and T2DM were managed at 0.25% (95% CI: 0.24–0.27%) and 3.68% (95% CI: 3.62–3.73%) of encounters respectively. By the end of the study, the proportion of encounters where prediabetes was managed was 2.3 times higher and for T2DM, 1.5 times higher. The proportion of prediabetes (55.9%, 95% CI: 53.9–57.8%) and T2DM (27.3%, 95% CI: 26.7–27.9%) management occasions where one or more hyperglycaemia-related tests were requested were relatively stable. However, differences in the types of tests were observed. For prediabetes, glucose tolerance tests were most common but from 2014/15, requests for HbA1c tests began to increase. For T2DM, HbA1c tests were most common, and requests for one or more glucose tests gradually declined.

**Conclusion:**

The observed 16-year annual trends align with the rising incidence of prediabetes and T2DM. GPs appeared to be strongly influenced by changes to the national insurance scheme and clinical guidelines for hyperglycaemia-related pathology testing. However, some GPs may have been pre-empting policy changes as there was also evidence of ‘unendorsed’ testing, notably for prediabetes, that warrants further investigation. The increasing proportion of encounters for prediabetes, coupled with a high proportion of management occasions where pathology was requested have substantial resource implications. Calls to lower the risk threshold for prediabetes screening therefore warrant an economic analysis. Ongoing, reliable, up-to-date data is needed to inform clinical practice guidelines and policy in Australia.

**Supplementary Information:**

The online version contains supplementary material available at 10.1186/s12875-022-01896-4.

## Introduction

The prevalence of Type 2 Diabetes Mellitus (T2DM) continues to rise globally. Current estimates are that around half a billion people worldwide have T2DM and this is projected to increase by at least 50% over the next 30 years [1]. In Australia, well over 1.2 million (4.9%) of the population have a diagnosis of diabetes, mostly T2DM [2, 3]. Another 1 in 6 Australians older than 25 years are likely to have prediabetes [4]. Primary medical care is the cornerstone of T2DM management in Australia. In 2015/16, around 4.0% of general practitioners’ (GP) clinical encounters involved T2DM management [5].

The Royal Australian College of General Practitioners (RACGP) in collaboration with Diabetes Australia, provides patient-centered recommendations aimed at optimizing diabetes diagnosis and management [6]. In 2016, glycated haemoglobin (HbA1c) was introduced as an alternative screening pathway to the standard fasting blood glucose (FBG) test that is followed by an oral glucose tolerance test (OGTT) if indicated [6]. In discussion with patients, GPs decide which screening pathway, FBG or HbA1c they will use.

Prediabetes – a state of glucose dysregulation that does not meet the diagnostic criteria of T2DM – is considered part of the continuum of glucose dysregulation culminating in T2DM. However, it is also a recognised clinical entity and a risk condition that is independently associated with an increased risk of the metabolic syndrome and cardiovascular disease [7, 8]. Studies have demonstrated that early identification of prediabetes when accompanied with lifestyle interventions, may reduce the risk of developing T2DM [9].

While T2DM is well defined, there is no agreed, universally recognised screening and diagnostic criteria for prediabetes [10–12]. Notably, the term ‘prediabetes’ is only used in the RACGP diabetes guidelines in the context of gestational diabetes, and instead ‘impaired fasting glucose’, ‘impaired glucose tolerance’, and ‘high risk HbA1c’ are used when referring to elevated results that do not meet the diagnostic criteria for T2DM [6]. In contrast, leading Australian non-medical primary care professional bodies have continued to use the term ‘prediabetes’ in their updated 2020 joint position statement on the screening and management of prediabetes in adults in primary care [12]. Along with FBG, HbA1c is now recommended as a first line screening test and a lower risk threshold is applied than that used for screening for T2DM. The addition of HbA1c and their use of the term prediabetes aligns with the American Diabetes Association guidelines [10].

Insulin, either fasting or with an OGTT, is another pathology test that GPs and medical specialists might use when screening and diagnosing prediabetes. Whilst these tests are not widely endorsed, since at least 2010 there have been calls to consider the role of insulin tests [13–15].

Given the current, and potential uses of these hyperglycaemia-related tests (FBG, HbA1c, OGTT and insulin) and their cost implications, it is important to document pathology referral activity by GPs in Australia within the broader picture of T2DM and prediabetes. A secondary analysis of publicly available Medicare Benefits Schedule (MBS) data found that between 2010 and 2019 pathology screening rates for T2DM doubled [16]. Limitations of the data included the MBS item numbers that bundle tests together, no information about who ordered the test, and little, if any information about the clinical circumstances.

Consequently, there remains a need to determine which hyperglycaemia-related pathology tests GPs in Australia use for managing prediabetes and T2DM, and how this may have changed over time relative to changes to Australian diabetes guidelines and MBS funding policies. The aim of this study was to investigate 16 years of GP encounters with patients aged 13 years and over (adolescents and adults) for the management of prediabetes and T2DM, and GP requests for hyperglycaemia-related pathology tests for the two conditions.

## Methods

### Study design

Analyses of data from the Bettering the Evaluation and Care of Health (BEACH) study, a national cross-sectional survey of GP clinical activity, with single-stage, cluster sampling of GPs, conducted from April 1998 to March 2016 [5].

### Setting, participants, data source & classification

The BEACH methods are described in detail elsewhere [5]. In brief, each year from April 1998 through to March 2016, the BEACH study involved ever-changing, random samples of approximately 1,000 GPs from across Australia, each of whom recorded details of 100 consecutive clinical encounters with consenting patients. At each encounter, participating GPs recorded deidentified clinical details including patient characteristics, up to four problems actively managed at the encounter (free text) and any management actions taken by the GP (directly linked to the problem being managed) that could include up to 5 pathology tests/batteries of test ordered (free text). The data were then coded by trained clinical coders using the Australian interface terminology ICPC-2 PLUS [17], which is classified according to the International Classification of Primary Care (ICPC-2) [18].

### Data analysis

Data collected in the last 16 years (April 2000 to March 2016) of the BEACH study were selected for the analysis. Only encounters with patients aged 13 years and over (adolescents and adults) were included. The ICPC-2 PLUS terms and codes rubric used to define prediabetes, T2DM, and the hyperglycaemia-related tests is outlined in Table 1. All point estimates were calculated as proportions, if an event could happen more than once (e.g. any glucose test) they were calculated as ‘at least one’ (e.g. at least one glucose test).

**Table 1:** Terminology

Terms used in this paper		ICPC-2 PLUS Code	Term/label	Clinical correlates
Prediabetes		A91011 A91012 A91035 A91016 A91028	Prediabetes abnormal glucose tolerance test impaired fasting glycaemia increased blood sugar insulin resistance	Prediabetes, impaired fasting glucose (IFG), or impaired glucose tolerance (IGT)
T2DM		T90^a^	Diabetes, non-insulin dependent	Type 2 Diabetes Mellitus, late onset diabetes, and diabetes not otherwise specified
Hyperglycaemia-related tests	Glucose-related tests	T34005 T34026 T34009 T34025 T34009	Glucose Glucose random Glucose challenging Glucose fasting Glucose tolerance	Blood glucose test, not specified Random blood glucose test Glucose challenge test Fasting blood glucose test Oral glucose tolerance test
	HbA1c test	T34010	HbA1c	Glycosylated haemoglobin test
	Insulin tests	T34019	Insulin	Insulin (fasting or random) test

^a^T90 is the ICPC-2 rubric for Diabetes, non-insulin dependent. This will include all ICPC-2 PLUS terms related to T2DM

Management occasions for prediabetes and T2DM are reported as proportions (percentage) of encounters. Over the 16 years of the study, there was a substantial increase in the number of problems managed per encounter, that in turn increased the chance of a management action occurring without any change in GP behaviour. Therefore, requests for hyperglycaemia-related tests are reported as proportions (percentage) of management occasions (i.e., when prediabetes (or T2DM) was a problem being managed) rather than proportion of encounters [5].

The types of patients seen, the problems managed, and treatments provided by the GP can be influenced by the characteristics of the GP. We accounted for the clustering of 100 encounters around each GP in the sample by using the survey means procedure in SAS v9.4 to calculate the intracluster correlation and adjust the 95% confidence intervals accordingly. Post-stratification weighting of encounter data was used to adjust for GP activity according to the number of MBS encounters each had claimed in the previous 12 months and for any minor differences in the age-sex distribution of participating GPs.

Statistically significant differences between point estimates were determined by non-overlapping 95% confidence intervals, which is a more conservative approach than the traditional alpha of 0.05 [19].

## Results

**Fig. 1:**
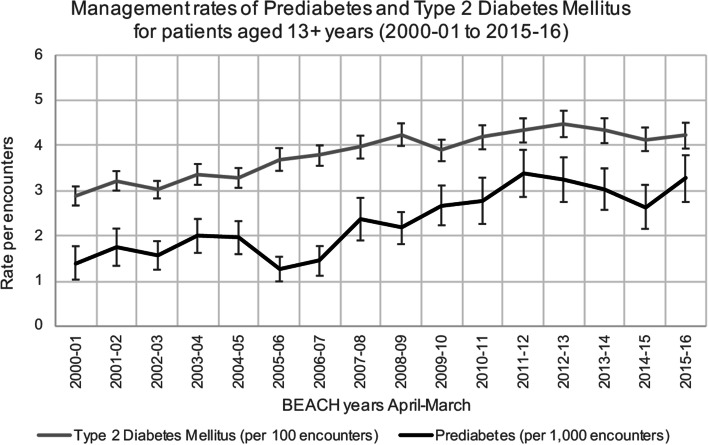
Management rates of Prediabetes and Type 2 Diabetes Mellitus for patients aged 13 + years (2000–01 to 2015–16). *Notes: Error bars signify 95% confidence interval*

Over the 16-year study period (April 2000 to March 2016), 15,679 GPs participated in the BEACH project, recording details of 1,387,190 encounters with patients aged 13 years or older. Substantially more encounters with patients involved T2DM management than prediabetes management (Fig. 1; Supplementary file 1). GPs managed T2DM in 50,979 (3.68%, 95% CI: 3.62–3.73%) of these encounters and prediabetes at 3,530 (0.25%, 95% CI: 0.24–0.27%) encounters. Statistically significant increases in the proportion of encounters where prediabetes was managed and where T2DM was managed were observed. In 2015–16, the proportion of encounters where T2DM was managed (4.22% of encounter (95% CI: 3.93–4.50%)) was 1.46 times higher than in 2000–01 (2.89% of encounters (95% CI: 2.67–3.10%)) Over the same period, the proportion of encounters where prediabetes was managed increased 2.33 times (0.14% of encounters in 2000–01 (95% CI: 0.10–0.18%) to 0.33% of encounters in 2015–16 (95% CI: 0.28–0.38%)).

**Fig. 2:**
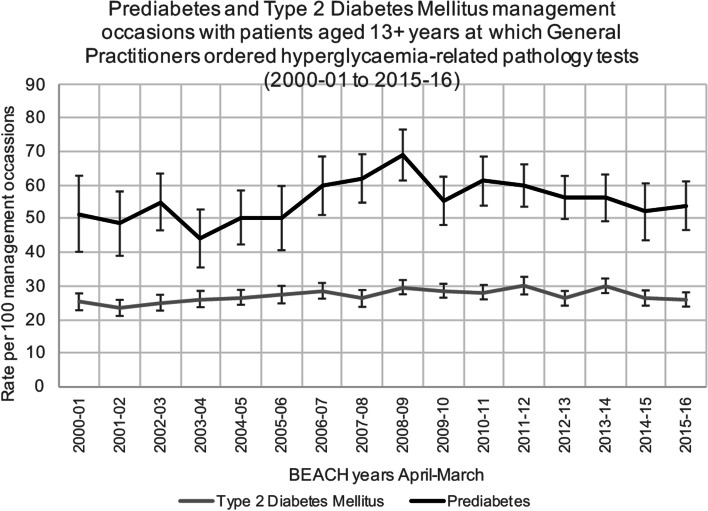
Prediabetes and Type 2 Diabetes Mellitus management occasions with patients aged 13 + years at which General Practitioners ordered hyperglycaemia-related pathology tests (2000–01 to 2015–16). *Notes: Error bars signify 95% confidence interval*

**Fig. 3:**
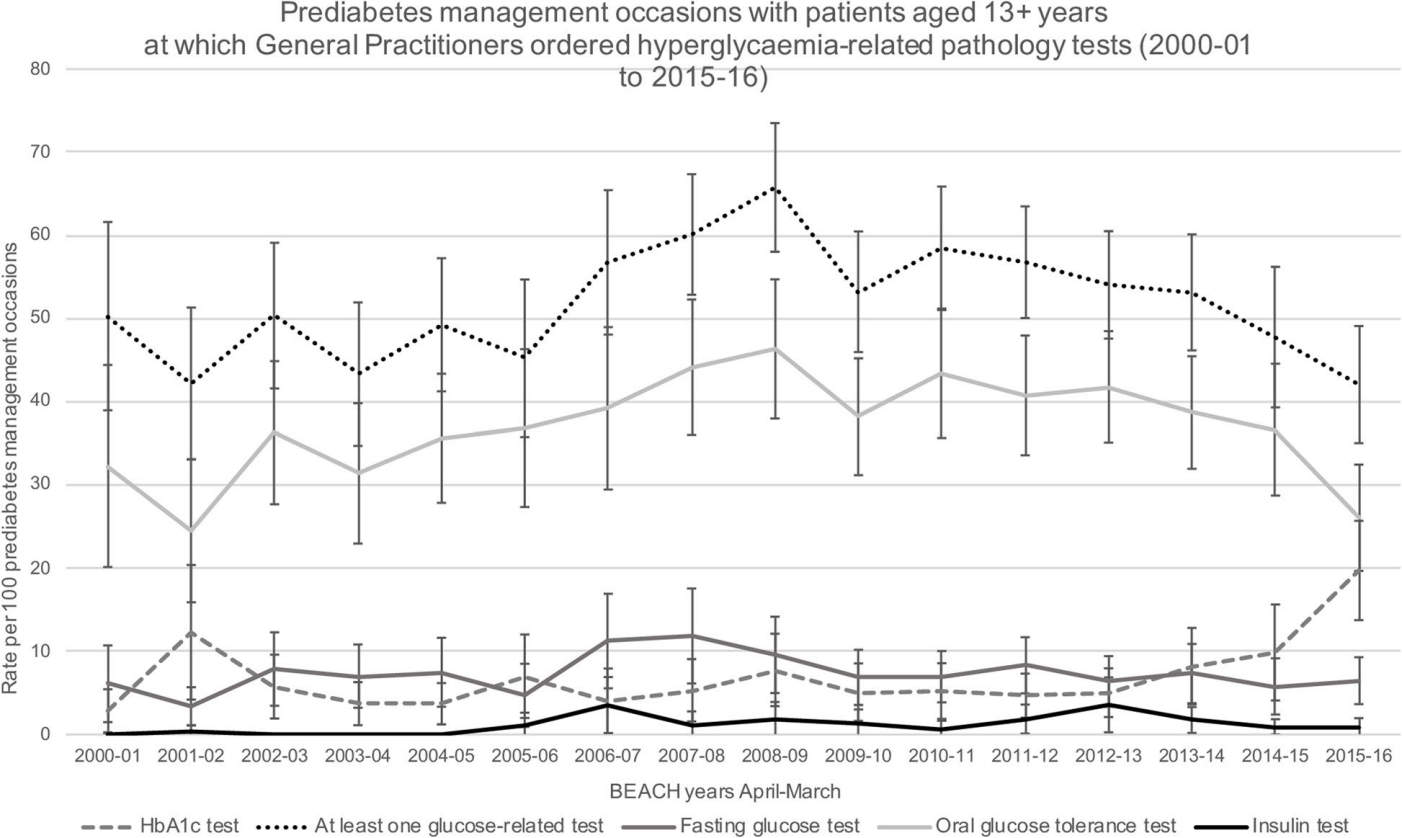
Prediabetes management occassions with patients aged 13 + years at which Generals Practitioners ordered hyperglycaemia-related pathology test (2000–01 to 2015–16). *Notes: Error bars signify 95% confidence interval*

**Fig. 4:**
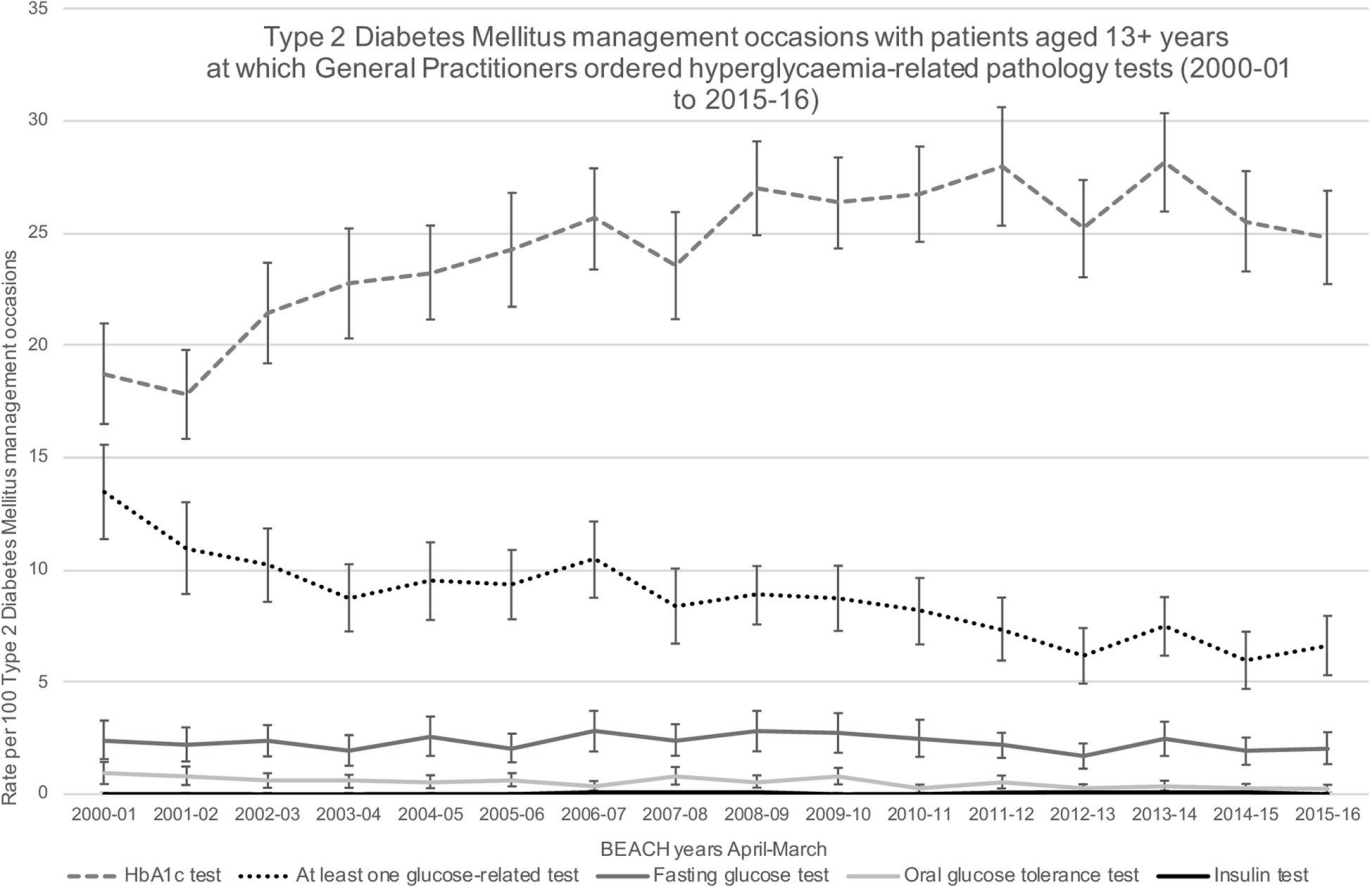
Type 2 Diabetes Mellitus management occasions with patients aged 13 + years at which Generals Practitioners ordered hyperglycaemia-related pathology test (2000–01 to 2015–16). *Notes: Error bars signify 95% confidence interval*

Over the 16-year study period, the proportion of prediabetes management occasions where at least one hyperglycaemia-related test was requested was 55.9% (95% CI: 53.9–57.8%) and for T2DM, 27.3% (95% CI: 26.7–27.9%). For both conditions, this was relatively stable across the 16 years of the study (Fig. 2, Supplementary file 1).However, when hyperglycaemia-related tests were considered separately for the management of prediabetes (Fig. 3, Supplementary file 1) and T2DM (Fig. 4, Supplementary file 1), differences were observed in both the proportions of the different hyperglycaemia-related tests requested and the annual trends.

Requests for one or more of the glucose-related tests at a prediabetes management occasion were by far the most common. Requests ranged from 42.2% (95% CI: 35.1–51.3%) of management occasions in 2001/02, to a peak of 65.8% (95% CI: 58.0–73.5%) in 2008/09. This trend was attributable to OGTTs that represented the bulk of the glucose-related tests. OGTTs were requested at 24.5% (95% CI: 15.9–33.1%) of management occasions in 2001/02 and peaked at 46.3% (95% CI: 54.7%-38.0%) in 2008/09. Except for requests for HbA1c tests in 2015/16, the proportion of prediabetes management occasions where the other tests were requested were significantly lower. The lowest proportion of requests for HbA1c tests was in 2001/02 (2.6% of management occasions, 95% CI: 0.3–5.4%). In 2015/16, the proportion of HbA1c requests rose to 19.7% of management occasions (95% CI: 13.7–25.7%). For FBG tests, proportions ranged between 3.4% (95%CI: 1.1–5.7%) of management occasions in 2001/02 to 11.9% (95% CI: 6.1–17.7%) of management occasions in 2007/08. Requests for insulin tests were the lowest. No insulin tests were requested for prediabetes management by any of the participating GPs in four of the 16 years of the study, and the highest proportion of requests was in 2012/13 (3.6% of management occasions, 95% CI: 2.5–6.9%) (Fig. 3).

For T2DM management, requests for HbA1c were significantly higher than for any of the other hyperglycaemia-related tests. There was a steady, significant increase in the proportion of HbA1c requests, starting at 18.7% (95% CI: 16.5–21.0%) of management occasions in 2000/01, increasing to 24.8% (95% CI: 22.7–26.9%) in 2015/16. Over the same timeframe, requests for any glucose-related test fell significantly from 13.5% (95% CI: 11.4–15.6%) to 6.6% (95% CI: 5.3–7.9%) of management occasions. Requests for both FBG (95% CI ranges 1.4% to 3.7% of management occasions) and OGTT (95% CI ranges 0.1% to 1.4% of management occasions) were substantially lower than for prediabetes management, and the OGTT was the least common glucose test for T2DM management. Pathology requests for insulin were also negligible, with proportions including the upper limit of the 95% CI remaining below 0.2% of management occasions (Fig. 4).

## Discussion

This study reports 16 years of hyperglycaemia-related pathology test ordering for the management of prediabetes and T2DM by GPs in Australia. During this time, 3.68% of GP encounters were for the management of T2DM and 0.25% were for prediabetes and the proportions increased by 1.5 and 2.3 times, respectively. For both conditions, the proportion of management occasions where one or more hyperglycaemic-related tests were requested remained relatively stable. However, significant differences were observed in the types of tests requested for the two conditions. For prediabetes management, OGTTs were most often requested, but from 2014/15, requests for HbA1c tests started to dramatically increase. For T2DM, HbA1c tests were most often requested, and the proportion of management occasions where HbA1c was requested steadily increased as requests for one or more glucose tests declined. Combined, these findings suggest that GP activity was influenced by nationally endorsed clinical practice guidelines for the use of pathology tests [6, 11, 20] and by national health insurance (MBS) funding [21]. However, ‘unendorsed’ and unfunded pathology tests were also requested.

The appropriate use of pathology tests is important for optimizing patient outcomes and there are substantial cost implications for both insurers and patients. It is reassuring then, that within the BEACH dataset used for this study, hyperglycaemia-related pathology testing for T2DM diagnosis and management generally aligned with the nationally endorsed recommendations [6, 11, 20]. Like other studies, there was also evidence that MBS funding of tests probably influenced GP clinical practice [22]. As such, both appear to be effective policy tools for promoting evidence-based medicine.

Notwithstanding, not all requests for pathology aligned with nationally endorsed guidelines. Some GPs appeared to be pre-empting national policy changes and were requesting HbA1c tests in the context of prediabetes management well before 2009, when some of the first calls were being made in Australia to add HbA1c tests for screening patients with an increased risk of developing T2DM [20], and also before the introduction of MBS funding near the end of 2014 [21]. Similarly, despite no national or international recommendations, insulin tests were requested by GPs, albeit infrequently and mostly for prediabetes management.

It is well recognised that clinical practice guidelines are often not followed for a wide range of reasons, including limited or emerging evidence, conflicting recommendations, clinical acumen and the need to tailor general recommendations to the individual patient [23–27]. For instance, only the evidence to inform HbA1c monitoring of long-term glucose control in T2DM was graded by the RACGP as high (A), and all other recommendations for pathology testing were graded as moderate (B) or low (C) [6]. Additionally, the frequency of prediabetes and T2DM risk assessments and requests for hyperglycaemia-related tests will vary depending on which Australian guideline the GP decides to follow [6, 11, 16]. For instance, whilst Australian guidelines consistently recommend GPs use the Australian Type 2 Diabetes Risk Assessment Tool (AUSDRISK) [28] to identify asymptomatic high risk patients, the specific recommendations differ [16]. The “Prediabetes: a position statement from the Australian Diabetes Society and Australian Diabetes Educators Association” recommends hyperglycaemia testing when the AUSDRISK score is between six and eleven, and to screen annually for scores higher than eleven [11]. In contrast, the RACGP guidelines only recommend hyperglycaemia testing every three years for scores higher than eleven [6]. The RACGP guidelines also recommend hyperglycaemia testing for individuals with specific clinical conditions or who identify as Aboriginal or Torres Strait Islander irrespective of their AUSDRISK score [6]. Other emerging evidence is also likely to influence screening decisions, such as calls for age-specific HbA1c reference intervals [29] and adding insulin tests for prediabetes screening, particularly for younger people and those at risk of metabolic syndrome [13–15, 30]. Further research is therefore warranted, both in the context of prediabetes and T2DM, and more broadly, to understand the factors that influence clinical decisions.

The findings also raise questions about potential resource implications of the 2020 prediabetes joint position statement for screening prediabetes in adults in primary care, as a lower risk threshold is applied, HbA1c is included, and more frequent testing is recommended [12]. During the 16-year study period, the proportion of GP encounters for prediabetes more than doubled and one or more hyperglycaemia-related tests were often requested at a management occasion. Since the end of the BEACH study in 2016, due to the changing demographics of the Australian population and rising rates of obesity, the prevalence of prediabetes and T2DM are continuing to rise [2, 12]. This in turn will lead to substantially more people undergoing a risk assessment, followed by more pathology testing not only for hyperglycaemia, but also for other conditions that individuals with prediabetes or T2DM have a higher risk of developing. Ongoing longitudinal data about the primary care activities and the clinical outcomes of patients at risk of prediabetes, T2DM and common comorbidities, is needed to help inform economic modelling of the potential costs and benefits of any proposed policy changes and whether additional MBS funding is indicated.

### Strengths and limitations of this study

The BEACH study is unique in the Australian setting. The very large data set, consistently collected over 16 years, coupled with the cluster analysis and weighted stratification approach has generated reliable, representative data to inform Australian healthcare planning and policies. The BEACH dataset allowed exploration of temporal changes in GP encounters and hyperglycaemia-related testing for the two conditions.

Limitations of the BEACH study include its cross-sectional study design, which did not allow us to determine how often individual patients consulted the GP each year or were being tested for the management of either condition, nor to explore the reasons GPs ordered tests not endorsed by national guidelines or funded by the MBS. Data were only available up to March 2016, the same year that substantial changes to the RACGP diabetes guidelines were made, so the full impact of RACGP endorsement of HbA1c for diagnosing T2DM could not be assessed. A maximum of five pathology tests, or suites of tests (e.g. liver function tests or lipid studies), could be recorded per encounter. Yet, it is common for GPs to order more tests, particularly when there is multimorbidity as is often the case with prediabetes and T2DM [3, 7]. Therefore, when selecting up to five tests, participating GPs might be inclined to first list tests that they perceived as most important and less contentious. As such, it is possible that insulin, and perhaps some prediabetes HbA1c testing, was underreported.

Limitations of this analysis included not investigating the number of tests requested for an individual patient encounter and whether there were any differences in the patient characteristics (e.g. age, gender, ethnicity, multimorbidity) for whom the different hyperglycaemia-related tests were requested. Another limitation was not investigating when hyperglycaemia-related tests were ordered for reasons other than T2DM and prediabetes, such as for metabolic syndrome, polycystic ovarian syndrome, other endocrine disorders, and routine health checks. Such tests for other morbidities were therefore not enumerated in this study.

## Conclusion

Annual trends in pathology requests for hyperglycaemia-related tests suggest that the clinical practice of GPs in Australia is strongly influenced by national guidelines and funding. Changes in Australian diabetes guidelines during the 16-year study corresponded with a significant increase in use of HbA1c and decline in glucose tests for the management of T2DM, and rising HbA1c testing for patients with prediabetes near the end of the study. While it is likely that glucose-related testing will remain the major mode of testing for prediabetes management, the impact of the recent 2020 joint position statement is uncertain. The BEACH study was completed in 2016, it has proved to be an essential information source for research, health system planning, and policy development. Similar projects are now urgently needed in Australia to ensure there is reliable, up-to-date, primary care data that can be used to inform government, industry, and not-for-profit organisations.

## Supplementary Information

**Additional file 1: Supplementary Table 1.** Proportion of encounters where Prediabetes and Type 2 Diabetes Mellitus (T2DM) were managed for patients aged 13+ years (2001-2 to 2015-16). **Supplementary Table 2.** Proportion of Prediabetes management occasions with patients aged 13+ years where General Practitioners requested hyperglycaemia-related pathology tests (2001-2 to 2015-16). **Supplementary Table 3.** Proportion of Type 2 Diabetes Mellitus (T2DM) management occasions with patients aged 13+ years where General Practitioners requested hyperglycaemia-related pathology tests (2001-2 to 2015-16).

## Data Availability

The dataset analysed during the current study is not publicly available as it contains health data with ethical and privacy restrictions placed on it. However, researchers with reasonable research questions may request analyses of these data if their questions are within the ethical guidelines set for the use of BEACH data. Data for the BEACH dataset can be requested from Dr Chris Harrison (christopher.harrison@sydney.edu.au).

## Abbreviations

T2DM: Type 2 diabetes mellitus
GP: General Practitioner
RACGP: Royal Australian College of General Practitioners
HbA1c: Glycated haemoglobin
FBG: Fasting blood glucose
OGTT: Oral glucose tolerance test
IFG: Impaired fasting glucose
MBS: Medicare Benefits Schedule
BEACH: Bettering the Evaluation and Care of Health
ICPC-2: International Classification of Primary Care
ICPC-2 PLUS: Australian interface terminology of the International Classification of Primary Care
