# Supplementary material for: Changes in hyperglycaemia-related testing for prediabetes and type 2 diabetes mellitus management: a prospective, cross-sectional survey of 16 years of general practice data from Australia
Source: BMC Prim Care. 2022 Nov 21;23:292. doi: 10.1186/s12875-022-01896-4 (PMC9680119; doi:10.1186/s12875-022-01896-4)
Supplement: Supplementary file 1 — Additional file 1: Supplementary Table 1. Proportion of encounters where Prediabetes and Type 2 Diabetes Mellitus (T2DM) were managed for patients aged 13+ years (2001-2 to 2015-16). Supplementary Table 2. Proportion of Prediabetes management occasions with patients aged 13+ years where General Practitioners requested hyperglycaemia-related pathology tests (2001-2 to 2015-16). Supplementary Table 3. Proportion of Type 2 Diabetes Mellitus (T2DM) management occasions with patients aged 13+ years where General Practitioners requested hyperglycaemia-related pathology tests (2001-2 to 2015-16). [file 12875_2022_1896_MOESM1_ESM.pdf]

BMC Primary Care: Supplementary file

Changes in hyperglycaemia-related testing for prediabetes and type 2 diabetes mellitus management: a prospective, cross-sectional survey of 16 years of general practice data from Australia. Andrew Leigh, Jennifer Hunter, Christopher Harrison, Helena Britt, Eugen Molodysky

Supplementary Table 1: Proportion of encounters where Prediabetes and Type 2 Diabetes Mellitus (T2DM) were managed for patients aged 13+ years (2001-2 to 2015-16)

Supplementary Table 2: Proportion of Prediabetes management occasions with patients aged 13+ years where General Practitioners requested hyperglycaemia-related pathology tests (2001-2 to 2015-16)

Supplementary Table 3: Proportion of Type 2 Diabetes Mellitus (T2DM) management occasions with patients aged 13+ years where General Practitioners requested hyperglycaemia-related pathology tests (2001-2 to 2015-16)

Supplementary Table 1: Proportion of encounters where Prediabetes and Type 2 Diabetes Mellitus (T2DM) were managed for patients aged 13+ years (2001-2 to 2015-16)

Year	2000-01	2001-02	2002-03	2003-04	2004-05	2005-06	2006-07	2007-08
Total no. of Encounters	87075	86311	88242	88395	84173	89887	82931	84425
Prediabetes (%)	1.40	1.75	1.57	2.00	1.96	1.27	1.45	2.37
lower 95% CI	*1.03*	*1.34*	*1.25*	*1.62*	*1.60*	*0.99*	*1.12*	*1.90*
upper 95% CI	*1.77*	*2.16*	*1.88*	*2.37*	*2.33*	*1.54*	*1.77*	*2.84*
T2DM (%)	2.88	3.22	3.02	3.36	3.28	3.69	3.78	3.97
lower 95% CI	*2.67*	*3.00*	*2.83*	*3.13*	*3.06*	*3.44*	*3.55*	*3.71*
upper 95% CI	*3.10*	*3.43*	*3.22*	*3.59*	*3.50*	*3.94*	*4.00*	*4.22*
Year	2008-09	2009-10	2010-11	2011-12	2012-13	2013-14	2014-15	2015-16
Total no. of Encounters	90384	86811	85083	87112	86529	85628	88231	85973
Prediabetes (%)	2.17	2.67	2.78	3.38	3.24	3.03	2.64	3.27
lower 95% CI	*1.82*	*2.24*	*2.26*	*2.86*	*2.75*	*2.58*	*2.16*	*2.75*
upper 95% CI	*2.53*	*3.11*	*3.29*	*3.91*	*3.74*	*3.49*	*3.13*	*3.79*
T2DM (%)	4.24	3.89	4.18	4.34	4.48	4.33	4.14	4.22
lower 95% CI	*3.99*	*3.65*	*3.92*	*4.07*	*4.18*	*4.05*	*3.88*	*3.93*
upper 95% CI	*4.49*	*4.13*	*4.45*	*4.60*	*4.77*	*4.60*	*4.40*	*4.50*

Changes in hyperglycaemia-related testing for prediabetes and type 2 diabetes mellitus management: a prospective, cross-sectional survey of 16 years of general practice data from Australia. Andrew Leigh, Jennifer Hunter, Christopher Harrison, Helena Britt, Eugen Molodysky

Supplementary Table 2: Proportion of Prediabetes management occasions with patients aged 13+ years where General Practitioners requested hyperglycaemia-related pathology tests (2001-2 to 2015-16)

Year	2000-01	2001-02	2002-03	2003-04	2004-05	2005-06	2006-07	2007-08
Total no. of prediabetes management occasions	118	155	179	186	180	134	136	212.00
At least one hyperglycaemia test (%)	51.50	48.57	55.02	44.15	50.40	50.20	59.84	62.01
lower 95% CI	40.19	39.01	46.58	35.51	42.38	40.66	51.14	54.80
upper 95% CI	62.81	58.13	63.46	52.78	58.42	59.75	68.54	69.21
At least one glucose related test (%)	50.27	42.20	50.34	43.32	49.24	45.23	56.74	60.10
lower 95% CI	*38.94*	*33.08*	*41.58*	*34.71*	*41.23*	*35.77*	*48.05*	*52.85*
upper 95% CI	*61.60*	*51.32*	*59.10*	*51.94*	*57.24*	*54.68*	*65.42*	*67.35*
Fasting glucose test (%)	6.09	3.39	7.87	7.02	7.48	4.70	11.24	11.85
lower 95% CI	*1.47*	*1.09*	*3.45*	*3.20*	*3.34*	*0.90*	*5.54*	*6.14*
upper 95% CI	*10.71*	*5.69*	*12.28*	*10.83*	*11.63*	*8.51*	*16.93*	*17.56*
Glucose tolerance test (%)	32.27	24.50	36.27	31.38	35.61	36.83	39.21	44.15
lower 95% CI	*20.13*	*15.89*	*27.68*	*22.96*	*27.87*	*27.35*	*29.47*	*36.03*
upper 95% CI	*44.40*	*33.11*	*44.86*	*39.80*	*43.35*	*46.30*	*48.94*	*52.28*
HbA1c test (%)	2.85	12.28	5.75	3.69	3.70	7.01	4.05	5.31
lower 95% CI	*0.26*	*4.18*	*1.92*	*1.10*	*1.20*	*1.99*	*0.16*	*1.57*
upper 95% CI	*5.44*	*20.39*	*9.59*	*6.28*	*6.19*	*12.03*	*7.93*	*9.06*
Insulin test (%)	0.00	0.37	0.00	0.00	0.00	1.07	3.53	1.04
lower 95% CI	*0.00*	*-0.36*	*0.00*	*0.00*	*0.00*	*-0.48*	*0.17*	*-0.68*
upper 95% CI	*0.00*	*1.10*	*0.00*	*0.00*	*0.00*	*2.62*	*6.90*	*2.77*
Year	2008-09	2009-10	2010-11	2011-12	2012-13	2013-14	2014-15	2015-16
Total no. of prediabetes management occasions	202	264	272	329	314	295	244	310
At least one hyperglycaemia test (%)	68.98	55.34	61.23	59.88	56.34	56.21	52.07	53.87
lower 95% CI	61.41	48.16	53.93	53.59	49.94	49.27	43.60	46.64

Changes in hyperglycaemia-related testing for prediabetes and type 2 diabetes mellitus management: a prospective, cross-sectional survey of 16 years of general practice data from Australia. Andrew Leigh, Jennifer Hunter, Christopher Harrison, Helena Britt, Eugen Molodysky

upper 95% CI	76.55	62.51	68.53	66.17	62.74	63.16	60.54	61.10
At least one glucose related test (%)	65.76	53.21	58.44	56.76	54.04	53.13	47.76	42.05
lower 95% CI	*58.02*	*45.96*	*51.02*	*50.05*	*47.58*	*46.15*	*39.31*	*35.03*
upper 95% CI	*73.49*	*60.46*	*65.87*	*63.47*	*60.49*	*60.10*	*56.20*	*49.08*
Fasting glucose test (%)	9.58	6.87	6.95	8.32	6.39	7.32	5.77	6.47
lower 95% CI	*4.98*	*3.55*	*3.86*	*4.94*	*3.35*	*3.75*	*2.40*	*3.65*
upper 95% CI	*14.17*	*10.19*	*10.04*	*11.70*	*9.43*	*10.89*	*9.15*	*9.28*
Glucose tolerance test (%)	46.34	38.20	43.41	40.75	41.79	38.70	36.65	26.08
lower 95% CI	*37.96*	*31.19*	*35.65*	*33.57*	*35.11*	*31.96*	*28.75*	*19.69*
upper 95% CI	*54.71*	*45.20*	*51.18*	*47.94*	*48.47*	*45.45*	*44.56*	*32.46*
HbA1c test (%)	7.75	5.08	5.22	4.68	5.02	8.20	9.86	19.72
lower 95% CI	*3.39*	*1.62*	*1.86*	*2.02*	*2.09*	*3.60*	*4.09*	*13.74*
upper 95% CI	*12.11*	*8.54*	*8.57*	*7.33*	*7.95*	*12.81*	*15.63*	*25.69*
Insulin test (%)	1.84	1.39	0.61	1.84	3.56	1.74	0.91	0.93
lower 95% CI	*-0.23*	*-0.23*	*-0.42*	*0.09*	*0.25*	*0.18*	*0.00*	*-0.09*
upper 95% CI	*3.91*	*3.01*	*1.65*	*3.59*	*6.87*	*3.29*	*1.82*	*1.96*

Changes in hyperglycaemia-related testing for prediabetes and type 2 diabetes mellitus management: a prospective, cross-sectional survey of 16 years of general practice data from Australia. Andrew Leigh, Jennifer Hunter, Christopher Harrison, Helena Britt, Eugen Molodysky

Supplementary Table 3: Proportion of Type 2 Diabetes Mellitus (T2DM) management occasions with patients aged 13+ years where General Practitioners requested hyperglycaemia-related pathology tests (2001-2 to 2015-16)

Year	2000-01	2001-02	2002-03	2003-04	2004-05	2005-06	2006-07	2007-08
Total no. of T2DM management occasions	2468	2705	2669	2886	2708	3170	2928	3186
At least one hyperglycaemia test (%)	25.30	23.50	25.04	26.13	26.64	27.45	28.60	26.30
lower 95% CI	*13.99*	*13.94*	*16.60*	*17.49*	*18.62*	*17.90*	*19.90*	*19.09*
upper 95% CI	*36.61*	*33.06*	*33.48*	*34.76*	*34.67*	*36.99*	*37.30*	*33.50*
At least one glucose related test (%)	13.46	10.96	10.21	8.75	9.49	9.33	10.45	8.38
lower 95% CI	*11.36*	*8.92*	*8.57*	*7.26*	*7.76*	*7.79*	*8.75*	*6.71*
upper 95% CI	*15.56*	*13.01*	*11.84*	*10.24*	*11.22*	*10.88*	*12.15*	*10.06*
Fasting glucose test (%)	2.42	2.22	2.38	1.95	2.58	2.06	2.81	2.42
lower 95% CI	*1.56*	*1.46*	*1.68*	*1.27*	*1.70*	*1.42*	*1.90*	*1.71*
upper 95% CI	*3.28*	*2.98*	*3.08*	*2.63*	*3.46*	*2.70*	*3.71*	*3.12*
Glucose tolerance test (%)	0.94	0.82	0.61	0.57	0.55	0.64	0.38	0.82
lower 95% CI	*0.45*	*0.41*	*0.28*	*0.28*	*0.27*	*0.35*	*0.17*	*0.43*
upper 95% CI	*1.43*	*1.23*	*0.94*	*0.86*	*0.84*	*0.94*	*0.59*	*1.21*
HbA1c test (%)	18.72	17.80	21.42	22.74	23.23	24.24	25.62	23.54
lower 95% CI	*16.48*	*16.48*	*16.48*	*16.48*	*16.48*	*16.48*	*16.48*	*16.48*
upper 95% CI	*20.96*	*20.96*	*20.96*	*20.96*	*20.96*	*20.96*	*20.96*	*20.96*
Insulin test (%)	0.00	0.00	0.03	0.02	0.00	0.00	0.06	0.06
lower 95% CI	*0.00*	*0.00*	*-0.03*	*-0.01*	*0.00*	*0.00*	*-0.01*	*-0.03*
upper 95% CI	*0.00*	*0.00*	*0.08*	*0.05*	*0.00*	*0.00*	*0.13*	*0.15*
Year	2008-09	2009-10	2010-11	2011-12	2012-13	2013-14	2014-15	2015-16
Total no. of T2DM management occasions	3816	3226	3464	3556	3571	3576	3561	3504
At least one hyperglycaemia test (%)	29.65	28.59	28.19	30.12	26.35	30.07	26.43	26.01
lower 95% CI	*22.08*	*21.42*	*20.89*	*23.84*	*19.94*	*23.12*	*17.96*	*18.78*

Changes in hyperglycaemia-related testing for prediabetes and type 2 diabetes mellitus management: a prospective, cross-sectional survey of 16 years of general practice data from Australia. Andrew Leigh, Jennifer Hunter, Christopher Harrison, Helena Britt, Eugen Molodysky

upper 95% CI	*37.22*	*35.77*	*35.49*	*36.41*	*32.75*	*37.01*	*34.90*	*33.24*
At least one glucose related test (%)	8.87	8.73	8.16	7.36	6.16	7.48	5.96	6.62
lower 95% CI	*7.56*	*7.28*	*6.68*	*5.96*	*4.93*	*6.17*	*4.69*	*5.30*
upper 95% CI	*10.17*	*10.18*	*9.64*	*8.76*	*7.40*	*8.78*	*7.24*	*7.94*
Fasting glucose test (%)	2.81	2.73	2.49	2.17	1.70	2.47	1.91	2.05
lower 95% CI	*1.91*	*1.85*	*1.66*	*1.61*	*1.14*	*1.70*	*1.31*	*1.34*
upper 95% CI	*3.71*	*3.61*	*3.32*	*2.74*	*2.26*	*3.23*	*2.52*	*2.76*
Glucose tolerance test (%)	0.57	0.80	0.25	0.54	0.25	0.38	0.26	0.24
lower 95% CI	*0.29*	*0.44*	*0.07*	*0.25*	*0.05*	*0.15*	*0.07*	*0.06*
upper 95% CI	*0.84*	*1.17*	*0.42*	*0.83*	*0.44*	*0.60*	*0.46*	*0.42*
HbA1c test (%)	26.98	26.33	26.72	27.96	25.19	28.14	25.52	24.79
lower 95% CI	*16.48*	*16.48*	*16.48*	*16.48*	*16.48*	*16.48*	*16.48*	*16.48*
upper 95% CI	*20.96*	*20.96*	*20.96*	*20.96*	*20.96*	*20.96*	*20.96*	*20.96*
Insulin test (%)	0.04	0.02	0.00	0.07	0.05	0.06	0.05	0.02
lower 95% CI	*-0.02*	*-0.01*	*0.00*	*-0.02*	*-0.02*	*-0.06*	*-0.05*	*-0.02*
upper 95% CI	*0.11*	*0.05*	*0.00*	*0.16*	*0.11*	*0.18*	*0.14*	*0.06*
